# Coccidioidomycosis among Scholarship Athletes and Other College Students, Arizona, USA^1^

**DOI:** 10.3201/eid1602.090918

**Published:** 2010-02

**Authors:** Nicole G. Stern, John N. Galgiani

**Affiliations:** University of Arizona Campus Health Service, Tucson, Arizona, USA (N.G. Stern); University of Arizona College of Medicine, Tucson (J.N. Galgiani)

**Keywords:** Community-acquired pneumonia, coccidioidomycosis, college students, athletes, public health, fungi, Arizona, valley fever, dispatch

## Abstract

To compare coccidioidomycosis case rates among groups of young adults in a disease-endemic region, we reviewed medical charts for serologic testing and coding. Case rates were higher for scholarship athletes than for other students and paralleled 5× more serologic testing. Our findings underscore the need to routinely test patients for coccidioidomycosis.

Coccidioidomycosis, known commonly as valley fever, is a fungal infection endemic to the southwestern United States (*1*). The illness most commonly caused is community-acquired pneumonia (*2**,**3*). Some researchers estimate that ≈50,000 infections are severe enough to warrant medical attention (*1**,**4*); however, reported infections represent less than a quarter of this estimate, creating uncertainty about the true impact of valley fever.

Scholarship athletes receive financial support for their educations in return for participation in intercollegiate varsity competition. Athletes comprise a precisely defined group and afford a special opportunity to calculate endemic risk for infection within a young adult population. We calculated case rates for scholarship athletes and then compared the rates with those for other students and persons of college age.

## The Study

The Campus Health Service at the University of Arizona is located on campus in Pima County. The average yearly enrollment during 1998–2006 of 36,000 was used for calculating case rates for all students (*5*). Scholarship athletes at the university total 475 annually.

Campus Health medical charts for January 1, 1998, through December 31, 2006, were reviewed for serologic testing for coccidioidomycosis. Charts were also reviewed for International Classification of Diseases, 9th edition (ICD-9), codes for coccidioidomycosis (114.0–114.5, 114.9) to identify students in whom the diagnosis was made by testing elsewhere. Athletes’ charts were identified based on billing information.

Most serologic testing, identified by its current procedural terminology (CPT) code, was performed by the Campus Health laboratory, which used PREMIER *Coccidioides* enzyme immunoassay (Meridian Diagnostics, Cincinnati OH, USA). Occasionally, ≈10% of the time, serum samples were tested by a single commercial laboratory, which used immunodiffusion testing for precipitin-type or complement fixing–type antibodies (*6*). Any positive result was considered diagnostic. Students tested multiple times for coccidioidomycosis were counted once, either in the first year for which results were positive or in the year first tested if all results were negative. Individual charts were reviewed for those students who did not have coccidioidal testing done at Campus Health and were included only if the diagnosis could be corroborated by outside laboratory documentation. A case of coccidioidomycosis was defined as one in which the diagnosis could be corroborated by positive coccidioidal serologic results, fungal culture, or histologic identification of spherules in biopsy specimens.

In addition, charts of all athletes who had coccidioidal infection were reviewed for demographic information, type of sport (indoors vs. outdoors), and extent of disease. Arizona state statistics were provided by the Arizona Department of Health Services. Not all statistics for all age ranges were available. All data were analyzed by using SPSS 11.0 for Windows (SPSS Inc, Chicago, IL, USA). Significance tests used the Fisher exact χ^2^. This study was approved by the University of Arizona Institutional Review Board and by the Campus Health Service Departmental Review Committee.

Of 2,754 student medical charts selected for review based on routine serologic testing for coccidioidomycosis or an ICD-9 code for coccidioidomycosis, 305 students were found to have coccidioidomycosis. Of these 305, a total of 297 had positive coccidioidomycosis results by serologic testing done at Campus Health and 8 were identified with diagnostic results from other laboratories. The resulting annual student case rate for all students was 94.1/100,000.

Annual rates for college students are 3–4× higher than available county and state rates for various age ranges (Table). The average age range of university students is 17–23 years. Because Pima County statistics includes cases reported to the state by Campus Health, actual differences between student rates and nonstudent Pima County rates for persons of similar ages would be even greater than represented here.

**Table:** Annual case rates per 100,000 for coccidioidomycosis in University of Arizona students and in selected age groups in Pima County, Arizona, and Arizona, USA, 1998–2006*

Year	University of Arizona students	Age group, y										
		15–24			15–19			20–24			25–29	
		Pima County	Arizona		Pima County	Arizona		Pima County	Arizona		Pima County	Arizona
1998	108											
1999	61											
2000	53	13.3	9.23									
2001	56	13.5	12.5									
2002	111	25.7	17.3									
2003	69	17.4	12.0									
2004	117	41	32.4		33.5	30.2		48.3	34.6		40.4	37.5
2005	147	53.2	30.1		39.7	23.6		66.3	36.7		64.9	38.4
2006	125	59.9	45.6		56.39	42.4		63.1	48.8		80.0	62.9

*The University of Arizona campus is located in Pima County. Blank cells indicate age ranges where composite statistics were not available.

Coccidioidal infections occurred in 16 scholarship athletes. The composite case rate, or annual incidence of coccidioidomycosis, was 374/100,000 (95% confidence interval [CI] 192–639) for scholarship athletes compared with 90/100,000 (95% CI 79–103) for other students (p<0.00001). In parallel, a much greater proportion (4.6%, 95% CI 3.9–5.4) of scholarship athletes were tested for coccidioidal antibodies than were other students (0.8%, 95% CI 0.77–0.84%) (p<0.000001). Infection for all but 1 athlete with disseminated infection was limited to the chest and/or skin, which resolved either untreated or after several months of antifungal therapy. The age distribution of scholarship athletes was the same as for other students, and only 1 of 16 athletes was from Arizona. Female athletes with coccidioidal infections comprised 56% of the total compared with 41% of all uninfected athletes (p = 0.31). For 81% of infected athletes, sports competition took place outdoors, whereas 88% of uninfected athletes competed outdoors (p = 0.43).

## Conclusions

A recent study in Arizona found that <13% of patients with community acquired pneumonia were tested for coccidioidomycosis (*7*). In contrast, Campus Health Service at the University of Arizona tested ≈50% of students with community acquired pneumonia for coccidioidomycosis (*8*,*9*). Consequently, proportionately higher than age-adjusted Pima County case rates are found. Similarly, the case rate of 374/100,000 for scholarship athletes, >4× higher than that for other students, is associated with a >5-fold increase in coccidioidal testing. Therefore, it would seem that most, if not all, of the increased case-rate for athletes can be accounted for by increased testing; little, if any, increased susceptibility can be attributed to increased exercise or athletic training. We did not evaluate why clinicians tested more scholarship athletes. We speculate that etiologic diagnoses for any illness that reduced physical performance were sought more frequently for scholarship athletes than for other students. However, we cannot entirely exclude the possibility that the higher rate of testing was to some extent a consequence of higher rates of pneumonia in scholarship athletes. In any instance, it would appear that more complete testing for community acquired pneumonia associated with valley fever does result in considerably higher estimates of case rates for this fungal infection.

In the past, skin test conversion rates have suggested an annual infection rate of ≈3,000/100,000 (*10*). A 2007 telephone survey conducted by the Arizona Department of Health Services of persons reported with new diagnoses of coccidioidomycosis found that the median length of residence in an endemic area was 17 years (*11*), further suggesting an average annual infection rate of ≈3%. In this context, rates for scholarship athletes fall well within these other estimates.

Case rates among scholarship athletes were actually underrepresented in outdoor sports, and the spectrum of disease severity was in line with that found in past studies of the student population as a whole (*8*). Many scholarship athletes come from regions where coccidioidomycosis is not endemic. Because fewer of these students had prior coccidioidal infection, the annual risk for the group as a whole would be slightly increased as shown in a previous report from the same college health group (*9*). Arizona state statistics show a marked increase with age in case rates, e.g., 163 cases/100,000 for persons >65 years of age. Our findings do not discount the possibility that immune responses of elderly patients differs from those of younger patients; disease in the elderly is more severe (*12*). However, our study suggests that at least an equal proportion of young adults have clinical illness from coccidioidal infection sufficient to seek medical evaluation.

Our findings address infection among college students, particularly scholarship athletes, and support a recommendation that student health clinicians and team physicians consider coccidioidomycosis as a possible etiology of illness in students who live in or have traveled recently to disease-endemic regions. More generally, our findings reinforce practice guidelines revised by the Infectious Diseases Society of America for management of community-acquired pneumonia to assist clinicians in evaluating patients with endemic exposure (*13*).
